# Management of Musculoskeletal Oligometastatic Disease in Breast Cancer

**DOI:** 10.3390/cancers17213578

**Published:** 2025-11-06

**Authors:** Kelly Kon-Liao, Josue Layme, Andrea Otero López-Lavalle, Marcos R. Gonzalez, Juan Pretell-Mazzini

**Affiliations:** 1 Facultad de Medicina, Universidad Peruana Cayetano Heredia, Lima 15102, Peru; kelly.kon@upch.pe; 2 Facultad de Medicina, Universidad Nacional Mayor de San Marcos, Lima 15102, Peru; josue.layme@unmsm.edu.pe; 3 Facultad de Medicina, Universidad Peruana de Ciencias Aplicadas, Lima 15102, Peru; u202016003@upc.edu.pe; 4 Tufts Medical Center Orthopaedic Residency Program, Tufts University School of Medicine, Boston, MA 02127, USA; marcos.gonzalez@tuftsmedicine.org; 5 Division of Orthopedic Oncology, Miami Cancer Institute, Baptist Health System South Florida, Plantation, FL 33324, USA

**Keywords:** breast cancer, bone disease, musculoskeletal disease, oligometastasis, treatment

## Abstract

Oligometastatic breast cancer represents an intermediate state between localized and disseminated disease with reasonable potential for clinical cure. Advancements in surgery, radiotherapy, and systemic therapy have improved prognosis. Due to the high prevalence of bone metastases, an increasing number of studies are evaluating new treatment strategies for oligometastatic bone disease. The decision to perform skeletal surgery is complex and depends on optimal patient selection. Major criteria include impending or pathologic long bone fractures, severe neurologic compromise, and an expected survival of over 3 months. Factors associated with improved survival include solitary bone metastases, preserved performance status, adequate surgical margins, absence of pathologic fracture, metachronous metastases, and ER-positivity status. Radiotherapy, especially SBRT, offers effective local control and palliation. Interventional radiology techniques such as percutaneous thermal ablation have also been described as potential treatment alternatives, particularly for fragile patients. Systemic treatment varies according to the tumor subtype. For HR+ and HER2 subtypes, a combination of endocrine therapy with CDK4/6 inhibitors may be considered. HER2+ patients are often treated with HER2-targeted therapies combined with chemotherapy. For triple-negative breast cancer, chemotherapy is the primary treatment. Bone-modifying agents are also recommended to maintain bone strength, prevent skeletal-related events, and reduce the need for additional interventions. Skeletal muscle metastases in breast cancer patients are rare and typically indicate advanced disease with poor prognosis. Treatment options include chemotherapy, radiotherapy, and surgical excision, but should be tailored to the patient’s clinical condition and prognosis.

## Simple Summary

Oligometastatic breast cancer refers to an intermediate state between localized and disseminated disease. Recent advances in surgery, radiotherapy, and systemic treatments are improving outcomes for these patients. However, choosing the right treatment can be challenging and must consider the patient’s overall condition and life expectancy. This article provides a comprehensive review of the literature to determine the optimal approach to the management of musculoskeletal oligometastatic disease in breast cancer. Indications for orthopedic surgery include pathologic fractures, neurologic compromise, and a long-expected survival. Favorable prognostic factors include solitary bone metastasis, preserved performance status, adequate surgical margins, absence of pathologic fracture, metachronous metastases, and ER-positivity status. As oligometastatic breast cancer still lacks standardized treatment strategies, further research is needed to develop treatment protocols suitable for broader clinical application.

## 1. Introduction

Breast cancer is the most common malignant tumor among women, comprising 24% of all malignancies in this population, with a 5-year survival rate of 32% for distant-stage disease [1,2]. A major factor contributing to the high mortality rate is the substantial risk of metastases, with bones being the most frequent site of metastatic spread [3]. Approximately 70% of people who die from breast cancer have evidence of bone metastatic disease at autopsy [4]. Conversely, skeletal muscle involvement is uncommon, and the literature regarding its prevalence is scarce [5]. Breast cancer subtypes are associated with different patterns of metastatic spread. For instance, HR+ subtypes are most likely to metastasize to the bone, while HER2+ subtypes have higher affinity for the brain, liver, and lungs. Triple-negative breast cancer (TNBC) has increased affinity for the lungs and brain [6,7].

Oligometastatic disease (OMD) is defined as the presence of no more than five metastases involving up to three organs, and is considered an intermediate stage between localized and disseminated disease [8,9]. The exact prevalence of oligometastatic breast cancer (OMBC) is unknown, but has been reported to be as high as 20% [10]. Advances in imaging techniques and locoregional treatments have made OMD increasingly recognizable, and growing evidence suggests that it is a distinct subgroup with a better long-term prognosis than metastatic breast cancer, offering a potential for clinical cure [11]. Notably, Milano et al. reported a 6-year overall survival (OS) of 47% among patients with OMBC treated with stereotactic body radiotherapy (SBRT) [12].

However, the outcomes of patients treated with curative intent are heterogeneous, indicating that OMD represents a spectrum of disease states with variable prognoses. To harmonize diagnostic criteria, the European Organisation for Research and Treatment of Cancer (EORTC) and the European Society for Radiotherapy and Oncology (ESTRO) have proposed a comprehensive classification system that conceptualizes OMD as an umbrella term encompassing all forms of limited metastatic disease according to biological and clinical characteristics [9,13]. Within this framework, genuine OMD refers to limited metastatic disease occurring without history of polymetastatic disease, whereas induced OMD describes cases with previous history of polymetastatic disease. Genuine OMD can be subclassified in de novo OMD, which refers to patients with first-time diagnosis of OMD, and repeat OMD, which occurs in patients with a previous history of OMD. Further subclassifications include oligorecurrence, oligoprogression, and oligopersistence, depending on whether the disease is detected during a treatment-free interval or active systemic therapy, and whether lesions are stable or progressing on current imaging [9].

Identifying these OMD states is clinically relevant, as they carry distinct prognostic implications and can guide individualized treatment strategies. In breast cancer, patients with de novo OMD generally show a more favorable prognosis and may benefit from aggressive local therapies such as surgery or SBRT with curative intent [14,15,16]. Oligorecurrent disease represents a more indolent tumor biology but is often associated with poorer survival outcomes due to limited curative locoregional treatment options. Selvarajan et al. reported a median OS of 74 months in de novo OMD compared to 22.7 months in oligorecurrent cases [17]. Oligoprogressive disease refers to acquired resistance to systemic treatment and is associated with poor survival outcomes [9]. In such cases, growing evidence supports the role of metastasis-directed therapy in restoring systemic treatment sensitivity and improving overall prognosis [18,19]. Recognizing these subtypes allows clinicians to tailor management strategies and to identify patients most likely to benefit from curative or disease-stabilizing approaches.

Among OMD presentations, bone involvement represents one of the most frequent and clinically significant patterns in breast cancer. Although metastatic bone disease is a heterogeneous entity, oligometastatic bone disease (OMBD) in breast cancer has better survival outcomes than generalized metastases [20]. Improved OS in patients with limited metastatic burden, along with advancements in surgery, radiotherapy, and systemic therapy, supports the multimodal approach recommended in the OMBC guidelines [21]. Historically, studies supporting SBRT have been largely retrospective or prospective non-randomized single-arm studies [22,23,24,25], and the role of surgery has been reported mainly in retrospective studies [22]. As aggressive metastasis-targeted therapies become more common, the growing number of randomized controlled trials on OMBD warrants further discussion. Our study aimed to provide a comprehensive review of the literature and ongoing trials to determine the optimal approach for managing musculoskeletal oligometastatic disease in breast cancer.

## 2. Overview of Treatment Options

The treatment of OMBD in breast cancer requires a multidisciplinary approach. Criteria for treatment selection should include tumor characteristics (location, size, histology, biological status), patient profile (health status, comorbidities, oncologic prognosis), number of metastatic sites, and time interval between diagnosis and development of metastases [26,27,28]. Due to the higher survival rate in OMD, the most aggressive treatment of this entity aims at local control, improved survival, progression-free survival (PFS), and symptom relief [28,29].

Currently, treatment options include surgery, radiotherapy, and systemic treatment (chemotherapy, targeted therapy, hormone therapy, immunotherapy, and bone-modifying agents) [27,30,31]. In most cases, a combination of local and systemic treatment is used to achieve good long-term effects [32,33].

## 3. Surgery

In the setting of bone metastases, surgery has been primarily reserved for vertebral metastases with spinal cord compression and for pathologic fractures of long bones [34]. Orthopedic treatment aims to decrease pain, increase mobility and functionality, achieve local tumor control, and prevent or stabilize pathologic fractures. The indication for surgical intervention in these cases is complex and varies according to the different management protocols of each institution. Surgical removal of metastatic bone tumors may not be necessary in some cases of breast cancer that respond to radiation or systemic therapy [35]. Ehne et al. consider the following criteria for patient selection that maximize the benefits of surgical intervention in patients with metastatic bone disease [34]: (I) presence of pathologic fracture or impending fracture in long bones, (II) severe neurologic compromise due to spinal metastatic disease, and (III) expected patient survival greater than three months.

Several algorithms addressing clinical staging, imaging, survival, and fracture risk prediction have been developed and are used as guides for making surgical decisions in people with metastatic bone disease [36,37,38,39]. However, heterogeneity in patients and the unpredictable nature of the tumor hinder their clinical application [40].

The axial skeleton is the most common site of metastasis in breast cancer, followed by long bones [41,42,43]. The site of metastasis is a key consideration in choosing the optimal surgical approach to ensure better survival and minimize the risk of recurrence. In solitary long bone metastases, resection followed by endoprosthetic reconstruction remains a generally accepted surgical option [34,44,45]. In axial skeleton metastases, radical tumor resection with reconstruction may be feasible, particularly in spinal lesions restricted to a single area, where the potential benefits may outweigh the surgical risks [46,47]. However, management strategies should also consider the number of metastases, the functional status of the patient and the specific anatomical location.

Despite retrospective studies indicating promising results for metastasectomy in OMD, the lack of randomized clinical trials leaves the true efficacy of surgical intervention unclear. Generally, candidates for bone metastasectomy should present with single or oligometastatic lesions and a life expectancy greater than 12 months [34,40,48,49]. However, there is currently no consensus regarding the precise indications for performing metastasectomy in patients with oligometastatic breast cancer involving the bone.

### 3.1. Surgical Treatment Options

Surgical options for appendicular bone metastases include intramedullary nailing (IMN), open reduction with internal fixation (ORIF), and endoprosthetic reconstruction (EPR) [40,50]. Less invasive procedures like IMN are typically preferred for patients with limited life expectancy, while en bloc resection and reconstruction with durable implants, such as prostheses, are recommended for those with longer expected survival [34,51].

**Figure 1:**
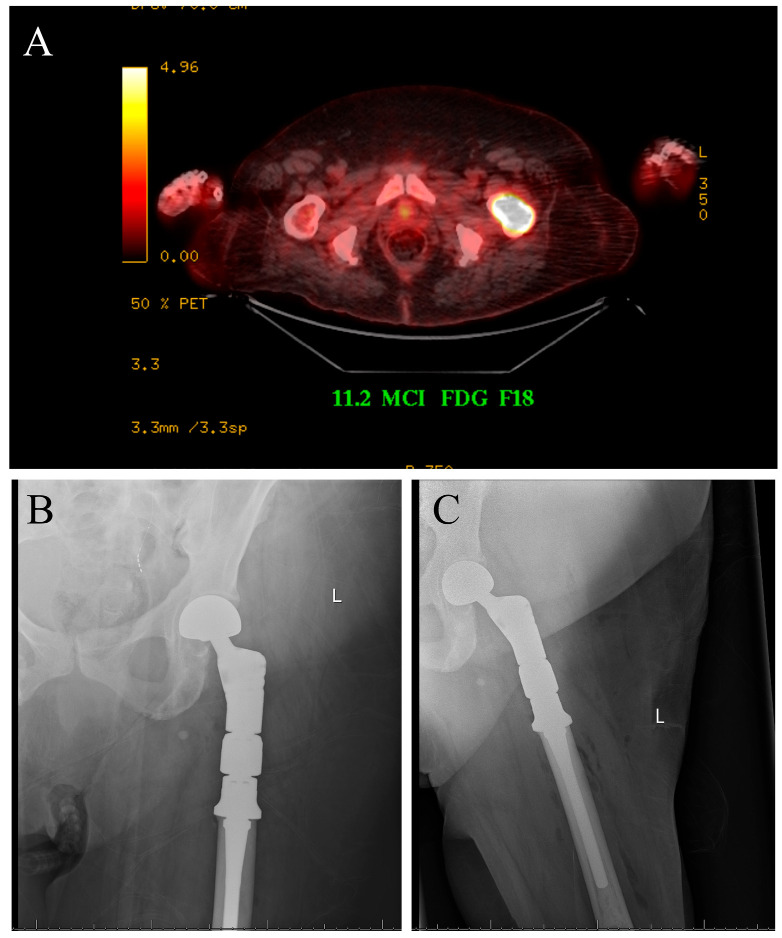
Seventy-five-year-old female with newly diagnosed breast cancer and pain with weight-bearing on the left hip. (**A**) Axial cut PET-CT showing solitary bone lesion biopsy proven breast cancer. (**B**,**C**) Patient underwent resection of left proximal femur and reconstruction with proximal femur megaprosthesis.

The case displayed in Figure 1 illustrates the surgical management of a solitary bone lesion on the hip by the senior author of this article.

Surgical treatment of impending or actual pathologic fractures due to metastases in long bones generally involves IMN due to the short operative time and early weight-bearing [49,50,51,52]. Lesions involving the femoral head or neck are typically treated with hip arthroplasty or EPR, as they offer a durable construct that allows for immediate weight-bearing [52,53,54]. In the peritrochanteric region, either internal fixation or arthroplasty is suitable [55], while PMMA augmentation is often used in the distal femur for added strength [34]. Humeral diaphyseal lesions are generally managed with IMN and are often reinforced with PMMA cement [56,57,58,59]. Metaphyseal humeral lesions may be treated with ORIF, which has shown adequate local tumor control and lower recurrence rates [60]. Proximal humeral lesions may require endoprosthetic replacement to preserve shoulder stability and function [59,61]. Distal humeral lesions are predominantly managed with plate fixation; however, more extensive resection with elbow reconstruction may be necessary for more complex fractures [62,63,64]. Tibial metastases, though less frequent, are addressed through EPR, IMN, or plate and screws osteosynthesis [65,66,67,68], with amputation reserved for advanced or non-salvageable cases [68,69].

Surgical management of spinal metastases ranges from minimally invasive procedures, such as vertebral augmentation, to more extensive interventions including radical tumor resection and reconstruction [46], with anterior or posterior approaches chosen based on tumor location, patient overall condition, and surgeon expertise [70,71,72,73,74,75]. Sternal metastases may benefit from aggressive resection and reconstruction with grafts or prosthesis implantation [76,77,78]. Surgery of pelvic metastases should also consider the location of the lesion within the bone. Periacetabular lesions often demand complex reconstruction to restore stability and function [79,80,81], while ileum and pubis lesions may not need reconstruction due to limited weight-bearing function [80]. Emerging minimally invasive procedures, such as percutaneous screw fixation or the use of photodynamic nails, are increasingly being used in the management of pelvic metastases, with growing evidence supporting good early functional outcomes, improved patient mobility, and early pain relief [80,82].

Skeletal muscle metastases are rare and very few reports exist on the subject [83,84]. Surgical resection may be helpful in carefully selected patients, such as those with painful lesions, long disease-free intervals, or radiation-resistant tumors [85,86,87,88]. Ultimately, surgical decisions are tailored to patient prognosis, anatomical site, and expected functional benefit.

### 3.2. Outcomes of Surgery in Oligometastatic Bone Disease in Breast Cancer

The benefits of surgery for bone metastases in breast cancer stem primarily from observational studies (Table 1). The 5-year OS after surgery for solitary bone metastases ranged from 39% to 56%, which is higher than that of metastatic breast cancer overall. However, survival is proportional to the number of lesions and can be as low as 8% in patients with multiple bone metastases [43,48,51]. Survival rates also differ by sociodemographic and economic factors, reflecting variations in tumor subtype prevalence, healthcare access, stage at diagnosis, and metastatic patterns. In a multivariate analysis, Ren et al. reported higher breast cancer–specific mortality among non-Hispanic Black women compared with non-Hispanic White women [89]. Similarly, on a global scale, survival rates vary by region, with poorer outcomes in low- and middle-income countries due to limited access to systemic therapies and specialized care [90].

The major issue with most of the available studies is the lack of a proper control group which limits the generalization of the results. Hankins et al. performed a retrospective review of 167 patients with breast cancer and bone metastases who received standard multimodality therapy (chemotherapy, radiotherapy, hormonal therapy, bone-directed therapy, and surgery) [91]. They compared the survival patterns of patients treated with and without surgery and found that non-operated patients had a higher median OS than operated patients, with 139.7 months and 88.9 months, respectively. However, surgically treated patients were more likely to have more aggressive lytic lesions which were often located in weight-bearing bones, this caused greater functional impairment and may have been associated with a lower overall survival. Also, the relatively small sample size and the combination of treatments received by patients limit the ability to identify differences in outcomes based on specific treatment approaches.

### 3.3. Prognostic Factors Related to Surgery

Several prognostic factors have been reported in the literature to improve survival after surgery in bone metastases. We present some of the most reported ones:

1. Solitary bone metastases [26,43,48,51];
2. Preserved performance status (Karnofsky score above 70) [95];
3. Adequate surgical margins (R0) [48];
4. Absence of pathologic fracture [48,92];
5. Metachronous metastases [91];
6. ER-positivity status [91,96,97].

Some risk factors for poor survival have also been reported (Table 2). Weiss et al. retrospectively reviewed 301 patients with breast cancer after surgical treatment of skeletal metastases [51]. They found age >60 years (HR 1.9, 95% CI 1.3–2.8, *p* = 0.002) and hemoglobin levels <110 g/L (HR 2.0, 95% CI 1.3–3, *p* = 0.001) as factors associated with increased risk of death, whereas impending fractures decreased postoperative risk of death (HR 0.4, 95% CI 0.2–1.0, *p* = 0.04). 14% of breast cancer patients who underwent surgical intervention for skeletal metastases required re-operation. The most common reasons were implant failure (*n* = 12), periprosthetic or stress fracture (*n* = 11), and local tumor progression (*n* = 8). However, these results are based on patients with various types of metastases (single skeletal, multiple skeletal, or generalized metastases) and may differ from those of patients with oligometastases.

Time of detection of metastases has also been recognized as an important predictor of survival, with synchronous and metachronous metastases representing two distinct patterns of metastases progression. Synchronous metastases are defined as metastases detected at the time of breast cancer diagnosis or within a maximum of 6 months from it, while metachronous metastases are identified after 6 months of initial breast cancer diagnosis [9]. Although no consensus exists in the literature regarding the time interval between the primary tumor diagnosis and the development of OMD for defining metachronous disease, a period of six months is frequently used. Hankins et al. reported median OS of 58.3 and 139.7 months for patients with synchronous and metachronous bone metastases, respectively [91]. Multivariable analysis confirmed that synchronous status was associated with inferior OS (HR 2.27, 95% CI 1.36–3.81, *p* = 0.002). Furthermore, they reported that surgical intervention of breast cancer bone metastases was not associated with a difference in OS in patients with *synchronous* bone metastases. The median OS of patients with synchronous metastases treated surgically was 58.3 months, compared with 59.9 months in patients with synchronous disease treated nonsurgically. In patients with metachronous metastases, the median OS for those treated surgically was 114.2 months, compared with 175.1 months for those treated nonsurgically. This study suggests that the timing of metastasis appearance is not a useful factor for patient selection for surgical intervention. Instead, decisions should be guided by lesion’s characteristics, location, size, and the patient’s performance status. The major limitation of this study is that it does not differentiate patients with oligometastases and disseminated disease; furthermore, the combination of treatments received by the patients and the retrospective nature of the study make it difficult to generalize the results.

## 4. Radiotherapy

Ablation therapies have been effective in treating metastases with minimal tumor spread, making them a potential alternative for managing oligometastases. Stereotactic ablative body radiotherapy (SABR) is increasingly being used for OMD because it offers a noninvasive approach that can be administered on an outpatient basis. SABR has also been demonstrated to be both feasible and well-tolerated in managing bone-involved OMBC, providing excellent local control. David et al. conducted a prospective trial involving 15 breast cancer patients with oligometastases who received a single fraction of SABR for all visible sites of disease [100]. They found that none of the patients experienced grade 3 or 4 radiotherapy-related toxicities, and only 27% of patients reported grade 2 toxicities.

Although systemic therapy is the standard of care for patients in a metastatic stage, cases of oligometastases can be treated in a metastasis-directed approach using minimally invasive surgical and ablative techniques, such as stereotactic ablative body radiotherapy or hypofractionated stereotactic radiation (HSRT). Milano et al. found that the 5-year and 10-year OS rates after HSRT were 83% and 75%, respectively, for patients with bone-only oligometastases, compared to 31% and 17%, respectively, for non-bone-only oligometastases [16]. Additionally, they emphasized that tumor burden, which includes both the volume and number of lesions, appears to influence the likelihood of recurrence.

### 4.1. Ongoing Clinical Trials

Various clinical trials are enrolling patients with OMBC, with radiotherapy being the main local treatment approach (Table 3) [23,100,101,102,103,104,105,106,107,108,109,110,111,112,113,114,115,116,117]. The number of metastases varies across studies, with a median ranging from 3 to 5 metastases. It is important to note that all patients received local ablative radiotherapy for progressing lesions. A major primary endpoint in these trials is the duration before a transition to systemic therapy is needed. Repeated local ablative therapy is usually only considered in the case of new oligoprogressive lesions [100].

It is important to highlight that the NRG-BR002 trial, a Phase II/III randomized controlled study, evaluated the addition of metastasis-directed treatments, including SBRT and/or surgical resection, to standard systemic therapy. The trial aimed to assess their impact on PFS and OS in patients with OMBC. However, the study did not demonstrate improvement in either PFS or OS and therefore did not advance to Phase III [106]. These findings underscore the need for further randomized controlled trials and comparative studies to better assess the efficacy and safety of metastasis-directed therapies, particularly SBRT, before such aggressive strategies can be systematically implemented in clinical practice.

### 4.2. Prognostic Factors Related to Radiotherapy

Some prognostic factors have also been reported in retrospective studies involving radiotherapy. Wijetunga et al. retrospectively reviewed 79 patients who underwent SABR for breast cancer oligometastases [15]. On multivariate analysis, they found that less than 5 years from breast cancer diagnosis to SABR (HR = 4.17, *p* = 0.003) was associated with shorter OS. Also, having TNBC was associated with worse PFS compared to HR+/HER2− breast cancer (HR = 2.80, *p* = 0.036).

Furthermore, retrospective studies have shown that prognosis worsens as the number of metastases increases. Steenbruggen et al. evaluated 3447 patients with synchronous metastatic breast cancer and reported that those with up to 3 metastases had an estimated 10-year OS rate of 14.9%, compared to 3.4% for patients with more than 3 metastases [118]. They additionally performed a multivariate analysis of 517 patients with OMBC and found that premenopausal (HR 0.37, *p* = 0.004) or perimenopausal status (HR 0.48, *p* = 0.03), along with local therapy of metastases (surgery and/or radiotherapy) (HR 0.57, *p* = 0.02) were associated with better OS. In contrast, lung metastases (HR 4.83, *p* < 0.001) and the absence of systemic treatment (HR 8.75, *p* = 0.002) were associated with worse OS. Although the study did not perform a subgroup analysis for patients with only skeletal metastases, 55.9% of patients included in the study had OMBC to the bone.

Other important predictors for good prognosis after SBRT include biological subtype (hormone receptor positive, HER2 negative), solitary metastasis, bone-only metastasis, and long-metastasis free interval [119]. However, further research with larger sample sizes and proper control groups is required to prove the benefits of SBRT in OMBC and define adequate selection criteria.

## 5. Interventional Radiology

Patients with OMBC are potentially curable due to very few metastasis sites, with limited potential for further spread. As a result, aggressive treatments aimed at cure, such as interventional radiology, confer chances to prolong survival in this intermediate cancer scenario [120,121]. Percutaneous thermal ablation (PTA) is a minimally invasive technique that may be performed on an outpatient basis targeting metastases in different locations with a relatively shorter time of recovery [122]. PTA encompasses various procedures such as radiofrequency ablation, microwave ablation, and cryotherapy. Barral et al. evaluated 79 patients with breast cancer oligometastases who underwent PTA with curative intent [123]. They reported no postoperative mortality and a morbidity rate of 15% (12 out 79 patients). The OS rates at 1 and 2 years were 98.3 and 95.5%, respectively. Disease-free survival rates at 1 and 2 years were 54.2 and 30.4%, respectively. Additionally, on multivariate analysis, they found that triple-negative histological subtype (HR 2.22, 95% CI 1.13–4.36, *p* = 0.02) and increased metastasis size (HR 2.43, 95% CI 1.22–4.82, *p* = 0.011) were associated with poorer disease-free survival following PTA. The study emphasized that PTA is a safe procedure with few and manageable complications, especially when compared to surgical metastasectomy in the oligometastatic setting and is frequently preferred for more fragile patients.

Ridouani et al. conducted a retrospective cohort study of 33 patients who underwent thermal ablation to either eradicate all evident sites of metastases or to achieve local control of metastases [122]. They found a median OS of 70 months and a 5-year OS of 55%, median PFS was 10 months and median time to local progression (TTP) was 11 months. The study also showed that ablation of 5 mm or more was associated with longer median TTP (13 vs. 5 months, *p* = 0.036), with no local tumor progression during the follow-up period. Additionally, Deschamps et al. conducted a retrospective analysis of patients who underwent thermal ablation of bone metastases with curative intent [121]. They reported a 1-year treatment completion rate of 67% and that favorable prognostic factors for successful local treatment include an oligometastatic and metachronous disease profile, small tumor size (less than 2 cm), and the absence of cortical erosions. This suggests that thermal ablation can also be indicated with curative intent.

## 6. Systemic Treatment

Selecting patients for systemic treatment involves evaluating specific characteristics, such as tumor characteristics, number and location of metastatic lesions, biological status of the tumor, patient profile, and the time interval between diagnosis and metastasis development [33,124]. Patients with OMBC tend to have one or two metastatic sites, primarily located in bone structures (53.2%), lymph nodes (19.7%), and liver (18.2%) [125]. Gogia et al. conducted an ambispective cohort study to describe the characteristics of 120 OMBC patients [126]. They found a variable distribution of hormone receptor statuses: 40% were ER and/or PR positive, 23.3% were ER/PR and HER2/neu positive, 17.5% were HER2/neu positive only, and 19.2% were triple-negative. Additionally, on multivariate analysis, they found that patients with hormone receptor-positive tumors (HR 0.46, *p* = 0.017), those with bone-only metastases (HR 0.54, *p* = 0.05), and those who underwent surgery of the primary tumor after neoadjuvant chemotherapy (HR 0.47, *p* = 0.013) had better PFS and clinical outcomes. This highlights the importance of systemic treatment, as it aims to slow the progression disease and manage symptoms [124].

Dürr et al. conducted a retrospective study to evaluate the effect of surgical therapy on a series of 70 patients with breast cancer and bone metastases [43]. Chemotherapy was used in 29 patients, of whom 9 received chemotherapy prior to surgery. They found that patients who underwent chemotherapy before surgery had worse prognosis than those who received chemotherapy afterward, likely due to more aggressive tumor behavior and resistance to systemic treatment. However, the heterogeneity of the patient population and the small sample size limit the generalizability of these findings. Studies evaluating the prognosis of bone metastases surgery following chemotherapy are still lacking.

**Figure 2:**
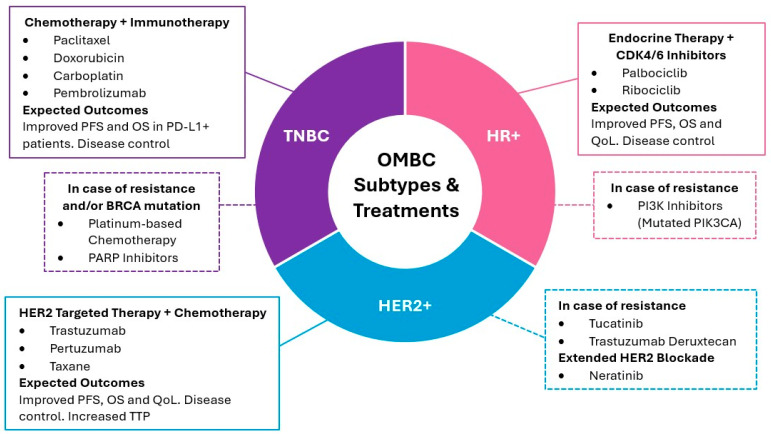
Systemic treatment strategies for OMBC according to biological subtype. The diagram outlines the primary treatment modalities for HR+, HER2+, and TNBC subtypes, including first-line therapies and options for managing resistance, and additional information on extended HER2+ blockade [33,116,124,125,126,130,131,132,133,134,135]. PFS: progression free survival; OS: overall survival; QoL: quality of life; TTP: time to progression.

Systemic therapy for OMBC varies based on the biological status of the tumor, aligning with specific molecular and genetic characteristics (Figure 2). For instance, Chmura et al. conducted a phase IIR/III trial to determine the efficacy of combining standard of care systemic therapy (SOC ST) with locoregional therapy (SBRT and/or surgical resection) as first line treatment of OMBC, compared to SOC ST alone [127]. They included 125 OMBC patients, most of whom presented with bone lesions, followed by metastases to the lung and liver, and predominantly exhibited an HR+/HER2- phenotype. First-line systemic therapy consisted of chemotherapy, endocrine, and targeted treatments in 27%, 76%, and 67% of patients, respectively. Although the trial did not meet the primary endpoint of PFS and no OS benefit was captured, locoregional therapy prevented new lesions in the index area with minimal high-grade toxicity [127,128]. For patients with OMBC, more aggressive locoregional treatments, combined with systemic therapy, can improve long-term survival, aligning with ESO-ESMO recommendations for treating patients who are highly sensitive to treatment [129].

### 6.1. Systemic Treatment Selection by Biological Status

Systemic treatment selection according to biological status has been reported in multiple clinical trials (Table 4). For HR+ and HER2- subtypes, combining endocrine therapy with CDK4/6 inhibitors like palbociclib or ribociclib has significantly improved progression-free and overall survival, especially in bone-only metastases [125]. In patients with a high tumor burden or rapid progression, adding everolimus or alpelisib may be beneficial, particularly in cases with PIK3CA mutations [33]. HER2-positive patients benefit from HER2-targeted therapies such as trastuzumab and pertuzumab, often combined with chemotherapy, with trastuzumab plus pertuzumab and a taxane as the standard first-line treatment [124,126]. In cases of progression, second-line therapies like trastuzumab emtansine (T-DM1) or trastuzumab deruxtecan show promising results [116,130]. For patients with TNBC, chemotherapy remains the standard treatment, with platinum-based regimens favored, particularly in patients with BRCA mutations. PD-1/PD-L1 inhibitors like pembrolizumab combined with chemotherapy have also shown survival benefits in selected TNBC patients [131]. In severe visceral involvement, immediate chemotherapy or systemic therapies are used to stabilize the patient [124].

### 6.2. Bone-Modifying Agents

Bone-modifying agents (BMAs) are essential in managing bone metastases from breast cancer, preventing skeletal-related events (SREs), reducing pain, and improving patients’ quality of life [149,150]. Bisphosphonates, such as zoledronic acid and pamidronate, inhibit osteoclast activity, preserving bone density and delaying SREs like pathological fractures, spinal cord compression, and the need for surgery or radiotherapy [151,152]. Zoledronic acid is particularly effective in reducing hypercalcemia of malignancy, a frequent complication in metastatic cancer, while also decreasing the need for radiotherapy when administered continuously for six months [151]. Additionally, it has demonstrated superior efficacy compared to pamidronate, reducing skeletal morbidity and delaying the time to the first SRE by up to 30% [152]. Zoledronic acid offers flexible dosing schedules, as it can be administered every 4 or 12 weeks without compromising treatment outcomes [152].

Denosumab, a fully human monoclonal antibody targeting the RANK/RANKL pathway, is a valuable alternative to bisphosphonates, particularly in patients with renal impairment where bisphosphonates might not be suitable [33,126,153]. By inhibiting osteoclast-mediated bone resorption, denosumab helps maintain bone strength and reduces the risk of multiple SREs, surpassing zoledronic acid in some clinical outcomes [152]. Its administration every 4 weeks via subcutaneous injection simplifies treatment, as it requires no renal monitoring, further enhancing its usability in clinical practice [152,153].

Current guidelines recommend both bisphosphonates and denosumab for managing bone metastases from breast cancer, emphasizing their role in reducing SREs, preserving bone strength, and minimizing the need for surgical or radiotherapeutic interventions [150]. Early initiation of bisphosphonate therapy, ideally within 3 months of surgery or 2 months of adjuvant chemotherapy, has been associated with better patient outcomes [150].

Combining BMAs with radiation therapy offers enhanced local control of osteolytic bone metastases, resulting in 75.0% bone reformation compared to 33.3% with BMAs alone [154]. This combination also accelerates symptom relief, with a median response time of 4 months versus 6 months for BMA-only treatment [154]. Moreover, patients responding to RT + BMA treatment show improved survival rates, with a 1-year overall survival rate of 83.1%, compared to 37.5% for non-responders [154].

## 7. Future Perspectives: Liquid Biopsies

Molecular advances such as liquid biopsies have emerged as promising tools for disease monitoring and treatment individualization in breast cancer. Circulating tumor DNA (ctDNA) consists of tumor-derived DNA fragments that carry specific mutations released into the bloodstream [155]. ctDNA analysis offers a minimally invasive method to characterize tumor genomic profiles, allowing early detection of residual disease and tumor-acquired resistance [156]. Notably, ctDNA can potentially detect disease relapse before clinical and radiological confirmation. Garcia-Murillas et al. reported a median lead time of 10.7 months between ctDNA detection and clinical relapse, anticipating relapse in all major breast cancer subtype [157].

In the OMBC setting, ctDNA analysis may refine treatment selection, as patients with undetectable ctDNA could represent optimal candidates for aggressive local therapies with curative intent [158]. Moreover, ctDNA provides insights into tumor heterogeneity and clonal evolution, supporting its role in tracking treatment response and predicting prognosis [159]. While prognostic biomarkers are not yet clinically available, a recent meta-analysis reported a significant association between ctDNA detection and poorer survival outcomes, underscoring its potential as a prognostic biomarker in metastatic breast cancer [160]. Integrating ctDNA analysis into OMBC management algorithms could therefore improve treatment individualization and guide future therapeutic decisions.

## 8. Conclusions

Oligometastatic bone disease is increasingly recognized as a tumor presentation in breast cancer patients that offers the potential for long-term tumor control. The standard of care for patients with oligometastatic breast cancer affecting the bones involves a multidisciplinary approach that includes radiotherapy, surgery, and systemic therapy. Orthopedic surgery interventions have shown favorable outcomes in a subgroup of patients, particularly in cases involving spinal metastases, pathologic fractures, and solitary or oligo skeletal disease, with positive neurological and functional outcomes. Radiation therapy plays a crucial role in both local control and palliation of the disease. Systemic treatment remains a key component of treatment for bone metastatic breast cancer, and its selection should be guided by the tumor’s biological, molecular, and genetic characteristics. Although current treatment strategies are increasingly multimodal and individualized, the lack of standardized treatment protocols and consensus guidelines remains a significant challenge, underscoring the need for further research and harmonization in clinical practice. As bone metastatic disease remains a significant cause of morbidity in breast cancer, future research must focus on enhancing diagnostic and treatment guidelines specifically for oligometastatic bone disease.

## Figures and Tables

**Table 1:** Outcomes of skeletal surgery in breast cancer patients with bone metastases.

Author	Patient (*n*)	Metastatic Site	Surgical Intervention	Outcomes Measures	Results
Wegener et al. [48]	115	Axial & Appendicular	vertebroplasty, decompression, endoprosthesis	complication rate mOS (single bone)	13% 65 months
Wedin et al. [53]	107	Axial & Appendicular	ostheosynthetic devices, endoprothesis	mOS reoperation rate	22 months 11%
Durr et al. [43]	70	Axial & Appendicular	endoprosthesis, spinal decompression, hip arthroplasty	1-year OS 2-year OS 5-year OS 10-year OS 5-year OS (single bone) local recurrence	59% 36% 13% 7% 39% 0%
Weiss et al. [51]	301	Axial & Appendicular	endoprosthesis, open reduction, internal fixation, spinal decompression	1-year OS 2-year OS 5-year OS complication rate reoperation rate	45% 27% 8% 25% 14%
Noguchi et al. [78]	9	Axial (sternum)	partial & total resection of sternum, lymph node dissection	mOS	30 months
Incarbone et al. [77]	19	Axial (sternum)	partial, subtotal & total resection of sternum	5-year OS (direct extension) 5-year OS (distant bone) local recurrence	48% 60% 0%
Hankins et al. [91]	76	Axial & Appendicular	NA	mOS	88.9 months
Mouraria et al. [92]	41	Appendicular (femur)	femoral prosthetic replacement, intramedullary nails and plates	mOS mortality	8.1 months 70%
Nathan et al. [93]	37	Axial & Appendicular	joint replacement, extremity fracture stabilization, spine decompression, amputation	mOS	13.5 months
Ratasvuori et al. [94]	364	Appendicular	endoprosthesis, nailing and plating procedures	mOS reoperation rate	12 months 5.9%

mOS: median overall survival; OS: overall survival; NA: not available.

**Table 2:** Risk factors for poor survival after skeletal surgery in breast cancer patients with bone metastases.

Author	Patient (*n*)	Metastatic Site	Risk Factors	Results
Durr et al. [43]	70	Axial & Appendicular	Tumor spread ^α^	RR 2.41 ^+^
			Duration of symptoms ^β^	RR 0.5 ^+^
Weiss et al. [51]	301	Axial & Appendicular	Age > 60 years	HR 1.9 ^+^
			Hemoglobin < 110 g/L	HR 2.0 ^+^
Hankins et al. [91]	76	Axial & Appendicular	Synchronous metastases	HR 2.27 ^+^
			Increased age at diagnosis	HR 1.03 ^+^
Mouraria et al. [92]	41	Appendicular (femur)	Proximal femur lesion	HR 2.97 ^+Φ^
			Diaphysis femur lesion	HR 3.32 ^+Φ^
			Drop 1 mg/dL hemoglobin	HR 1.26 ^+^
			Increase 1 mg/dL creatinine	HR 3.65 ^+^
			Presence of fracture	HR 3.13 ^+^
Sciubba et al. [97]	87	Axial (spine)	Cervical lesion	RR 2.1 ^++^
			Negative estrogen receptor	RR 3.7 ^+^
Shehadi et al. [98]	87	Axial (spine)	Instrumentation of ≥5 spinal levels	OR 7.2 ^+^
Yao et al. [99]	54	Axial (spine)	Hormone therapy insensitivity	HR 1.68 ^+^
			Brain metastases	HR 3.55 ^+^

HR: hazard ratio; RR: rate ratio; ^+^ *p*-value < 0.05; ^++^ *p*-value = 0.06; ^α^ solitary osseous, multiple osseous, visceral; ^β^ ≤3 months, >3 months; ^Φ^ subtrochanteric reference.

**Table 3:** Clinical trials of radiation therapy for oligometastatic breast cancer patients.

Identifier	Primary Tumor	Design	Patient (*n*)	No. of Metastases	Metastatic Site	Intervention	Outcomes Measure	Results
CRO 2012-47 [23]	Breast	Prospective Phase II Trial	54	1–5	Mixed (60 bone)	SABR or IMRT	1-year PFS 2-year PFS 2-year LC 2-year OS	75% 53% 97% 95%
U1111-1154-1830 [100]	Breast	Prospective Clinical Trial	15	1–3	Bone	SABR	2-year LPFS 2-year DPFS	100% 67%
NCT01446744 [101]	Mixed	Phase II Randomized Trial	99 (18 breast)	1–5	Mixed (65 bone)	arm 1: SOC arm 2: SOC + SABR	5-year OS	17.7% (arm 1) vs. 42.3% (arm 2)
							5-year PFS	not reached (arm 1) vs. 17.3% (arm 2)
NCT02581670 [102]	Breast	Phase II Non-randomized Prospective Trial	64	1–4	Lung, Liver	SBRT	3-year LC mOS 3-year OS mDMFS 3-year DMFS	91% 16.5 mo 51.9% 8.3 mo 16%
NCT03862911 [103]	Mixed	Phase III Randomized Trial	330	1–3	NA	SABR vs. SOC	OS	pending (recruiting)
NCT03721341 [104]	Mixed	Phase III Randomized Trial	204	4–10	NA	SABR + SOC vs. SOC	OS	pending (active, not recruiting)
NCT04495309 [105]	Breast	Randomized Controlled Trial	564	1–5	NA	RT + SOC vs. SOC	PFS QoL	pending (unknown status)
NCT02364557 [106]	Breast	Phase II/III Randomized Trial	129	1–4	Mixed	arm 1: SOC arm 2: SOC + RT/Surgery	PFS	23 mo (arm 1) vs. 19.5 mo (arm 2)
							OS	36-mo OS: 71.8% (arm 1) vs. 68.9% (arm 2)
NCT03808337 [107]	Breast, Lung	Phase II Randomized Trial	145	1–5	NA	SABR + SOC vs. SOC	PFS	pending (recruiting)
NCT03750396 [108]	Breast	Phase II Single Arm	110	1–2	Mixed	RT/RFA/Surgery + SOC	PFS	pending (unknown status)
NCT04698252 [109]	Breast	Phase II Randomized Trial	74	1–4	Mixed	RT/RFA/Surgery + SOC vs. SOC	PFS	pending (recruiting)
NCT04424732 [110]	Breast	Prospective Single Arm	50	1–3	Bone	SBRT	PFS OS	pending (recruiting)
NCT02089100 [111]	Breast	Phase III Randomized Trial	280	1–5	Mixed	SABR + SOC vs. SOC	PFS	pending (recruiting)
NCT06144346 [112]	Breast	Phase III Randomized Trial	150	1–5	NA	SBRT + SOC vs. SOC	PFS OS	pending (recruiting)
NCT04646564 [113]	Breast	Phase III Randomized Trial	170	1–5	NA	RT + SOC vs. SOC	PFS	pending (recruiting)
NCT06135714 [114]	Breast	Phase III Randomized Trial	340	1–3	NA	RT/Surgery + SOC vs. SOC	OS	pending (recruiting)
NCT02759783 [115]	Breast, Lung, Prostate	Phase II/III Randomized Trial	245	1–3	Mixed	SABR + SOC vs. SOC	PFS	pending (unknown status)
NCT05377047 [116]	Breast	Phase III Randomized Trial	345	1–5	Mixed	SABR + SOC vs. SOC	PFS OS	pending (recruiting)
NCT03143322 [117]	Breast, Prostate, NSCLC	Phase III Randomized Trial	196	1–5	Bone	SABR + SOC vs. SOC	PFS	pending (active, not recruiting)

OS: overall survival; SOC: standard of care; SABR: stereotactic ablative body radiotherapy; SBRT: stereotactic body radiotherapy; IMRT: intensity modulated radiotherapy; PFS: progression free survival; LC: local recurrence; DMFS: distant metastasis-free survival; NA: not available; mo: months.

**Table 4:** Outcomes of systemic treatment selection by biological status in metastatic breast cancer patients.

Author	Study Type	Patient (*n*)	Biological Status	Metastatic Site	Systemic Treatment	Outcomes Measure	Results
Cassier et al. [136]	Phase III Randomized Trial	210	74% were HR+	Mixed (20 bone)	Doxorubicin + Docetaxel (arm D) vs. Doxorubicin + Paclitaxel (arm P)	PFS mOS	8.7 (arm D) vs. 8.0 mo (arm P) 21.4 (arm D) vs. 27.3 mo (arm P)
Sledge et al. [137]	Phase III Randomized Trial	739	45% were ER+	Mixed (130 bone)	Doxorubicin (arm D) vs. Paclitaxel (arm P) vs. Doxorubicin + Paclitaxel (arm AT)	Response rate	36% (arm D) vs. 34% (arm P) vs. 47% (arm AT) 5.8 (arm D) vs. 6.0 (arm P) vs. 8.0 mo (arm AT)
						TTF	
Schmid et al. [138]	Phase III Randomized Trial	902	TNBC	Mixed (286 bone)	Atezolizumab + Nab-paclitaxel (arm A) vs. Placebo + Nab-paclitaxel arm B)	PFS	7.2 (arm A) vs. 5.5 mo (arm B)
Finn et al. [139]	Phase III Randomized Trial	666	HR+/HER2-	Mixed (151 bone)	Palbociclib + Letrozole (arm A) vs. Placebo + Letrozole (arm B)	PFS	24.8 (arm A) vs. 14.5 mo (arm B)
Hortobagyi et al. [140]	Phase III Randomized Trial	668	HR+/HER2-	Mixed (147 bone)	Ribociclib + Letrozole (arm A) vs. Placebo + Letrozole (arm B)	PFS	25.3 (arm A) vs. 16.0 mo (arm B)
André et al. [134]	Phase III Randomized Trial	341	HR+/HER2- with PIK3CA mutation	Mixed (77 bone)	Alpelisib + Fulvestrant (arm A) vs. Placebo + Fulvestrant (arm B)	PFS	11.0 (arm A) s 5.7 mo (arm B)
Swain et al. [141]	Phase III Randomized Trial	808	HER2+	Mixed	Pertuzumab + Trastuzumab + Docetaxel (arm A) vs. Placebo + Trastuzumab + Docetaxel	PFS mOS	18.7 (arm A) vs. 12.4 mo (arm B) 56.5 (arm A) vs. 40.8 mo (arm B)
Cortes et al. [142]	Phase III Randomized Trial	847	TNBC	Mixed (255 bone)	Pembrolizumab + Chemotherapy (arm A) vs. Placebo + Chemotherapy (arm B)	PFS mOS	7.5 (arm A) vs. 5.6 mo (arm B) 17.2 (arm A) vs. 15.5 mo (arm B) ^+^
Diéras et al. [143]	Phase III Randomized Trial	991	HER2+	Mixed	Trastuzumab emtansine (arm A) vs. Capecitabine + Lapatinib (arm B)	PFS mOS	9.6 (arm A) vs. 6.4 mo (arm B) 29.9 (arm A) vs. 25.9 mo (arm B)
Arpino et al. [144]	Phase II Randomized Trial	258	HR+/HER2+	Mixed	Pertuzumab + Trastuzumab + Aromatase inhibitor (arm A) vs. Trastuzumab + Aromatase inhibitor (arm B)	PFS mOS	20.6 (arm A) vs. 15.8 mo (arm B) 60.2 (arm A) vs. 57.2 mo (arm B)
Huober et al. [145]	Phase III Randomized Trial	57	HR+/HER2+	Mixed (34 bone)	Letrozole alone (arm A) vs. Letrozole + Trastuzumab (arm B)	TTP CBR	3.3 (arm A) vs. 14.1 mo (arm B) 39% (arm A) vs. 65% (arm B)
Johnston et al. [146]	Phase III Randomized Trial	355	HR+/HER2+	Mixed (172 bone)	Lapatinib + Trastuzumab + Aromatase inhibitor (arm A) vs. Trastuzumab + Aromatase inhibitor (arm B) vs. Lapatinib + Aromatase inhibitor (arm C)	PFS	11.0 (arm A) vs. 5.7 (arb B) vs. 8.3 mo (arm C)
Wesolowski et al. [147]	Phase Ib Non-randomized Trial	41	HR+/HER2-	Mixed (26 bone)	Gedatolisib + Palbociclib + Letrozole	PFS mOS	48.4 months 77.3 months
Bardia et al. [148]	Phase III Randomized Trial	468	TNBC	Mixed (103 bone)	Sacituzumab govitecan (arm A) vs. Single-agent chemotherapy (arm B)	PFS mOS	5.6 (arm A) vs. 1.7 mo (arm B) 12.1 (arm A) vs. 6.7 mo (arm B)

HR: hormone receptor; ER: estrogen receptor; TNBC: triple-negative breast cancer; HER2: human epidermal growth factor 2; PFS: progression free survival; mOS: median overall survival; TTF: time to treatment failure; TTP: time to progression; CBR: clinical benefit rate; mo: months; ^+^ significance not tested.
